# Metagenomic insights into mixotrophic denitrification facilitated nitrogen removal in a full-scale A2/O wastewater treatment plant

**DOI:** 10.1371/journal.pone.0250283

**Published:** 2021-04-15

**Authors:** Shulei Liu, Yasong Chen, Lin Xiao

**Affiliations:** School of the Environment, State Key Laboratory for Pollution Control and Resource Reuse (SKL-PCRR), Nanjing University, Nanjing, China; The University of Akron, UNITED STATES

## Abstract

Wastewater treatment plants (WWTPs) are important for pollutant removal from wastewater, elimination of point discharges of nutrients into the environment and water resource protection. The anaerobic/anoxic/oxic (A2/O) process is widely used in WWTPs for nitrogen removal, but the requirement for additional organics to ensure a suitable nitrogen removal efficiency makes this process costly and energy consuming. In this study, we report mixotrophic denitrification at a low COD (chemical oxygen demand)/TN (total nitrogen) ratio in a full-scale A2/O WWTP with relatively high sulfate in the inlet. Nitrogen and sulfur species analysis in different units of this A2/O WWTP showed that the internal sulfur cycle of sulfate reduction and reoxidation occurred and that the reduced sulfur species might contribute to denitrification. Microbial community analysis revealed that *Thiobacillus*, an autotrophic sulfur-oxidizing denitrifier, dominated the activated sludge bacterial community. Metagenomics data also supported the potential of sulfur-based denitrification when high levels of denitrification occurred, and sulfur oxidation and sulfate reduction genes coexisted in the activated sludge. Although most of the denitrification genes were affiliated with heterotrophic denitrifiers with high abundance, the *narG* and *napA* genes were mainly associated with autotrophic sulfur-oxidizing denitrifiers. The functional genes related to nitrogen removal were actively expressed even in the unit containing relatively highly reduced sulfur species, indicating that the mixotrophic denitrification process in A2/O could overcome not only a shortage of carbon sources but also the inhibition by reduced sulfur of nitrification and denitrification. Our results indicate that a mixotrophic denitrification process could be developed in full-scale WWTPs and reduce the requirement for additional carbon sources, which could endow WWTPs with more flexible and adaptable nitrogen removal.

## Introduction

With the increasing realization of the impacts of excess nitrogen (N) discharge on the environment and human health, N effluent regulations have become increasingly stringent worldwide. Until now, the biological process of nitrification/denitrification has been the most prevalent wastewater treatment plant (WWTP) used to remove N from wastewater. Normally, the ratios of COD (chemical oxygen demand)/TN (total nitrogen) and BOD (biological oxygen demand)/TN are required to be higher than 15 and 8, respectively, to supply a sufficient carbon source for the traditional nitrification/denitrification process [1,2]. To ensure N removal, external organics are often required, and the additional consumption of carbon and energy has become a challenge in some WWTPs.

As sustainable wastewater treatment becomes a priority, autotrophic denitrification processes based on sulfur oxidation have become increasingly popular [3–5]. Sulfur-based autotrophic denitrification, such as the sulfate reduction autotrophic denitrification nitrification integrated (SANI^®^) process and sulfur-limestone autotrophic denitrification (SLAD), has been comprehensively studied [3,5–8]. In these processes, chemolithotrophic sulfide-oxidizing denitrifying bacteria (SONB) (*Thiobacillus* sp., *Sulfurimonas denitrificans*, *Beggiatoa* sp., and *Thiothrix* sp.) and heterotrophic sulfide-oxidizing denitrifiers (*Thauera*-like taxa, *Azoarcus*, *Pseudomonas*, and *Dechloromonas*) cooperate in the processes of N removal [3,5,9–14]. Moreover, sulfate reducing bacteria (SRB), such as *Desulfobacteraceae*, *Desulfonema*, and *Thermotogaceae*, in activated sludge (AS), which convert sulfate to sulfur, sulfide, and poly-S, could cooperate with SONB and enhance N removal by providing electron donors [3,8,15].

Normally, studies of sulfur-based denitrification have focused on wastewater containing high concentration of sulfate, such as saline sewage [3,16]. In fact, some inland WWTPs, especially WWTPs located in industrial parks, also treat influent containing high sulfate concentrations due to the high concentration of sulfate in industrial wastewater [17,18], which might boost the development of sulfur-based denitrification. In our previous study, we analyzed the performance of a full-scale WWTP (referred to hereafter as the YXM WWTP) based on an anaerobic/anoxic/oxic (A2/O) process in an industrial park in Yixing, China, and found that the WWTP could achieve efficient N removal under a low ratio of COD/TN [19]. In the YXM WWTP, approximately 40% of the influent was collected from an industrial park, and the average concentration of SO_4_^2-^-S in the inlet was over 100 mg L^-1^, which could provide the sulfate needed in mixotrophic denitrification. Moreover, organic carbon, N and sulfate coexist in actual wastewater. Mixotrophic denitrification, which integrates the advantages of heterotrophic and autotrophic denitrification, is promising in removing N, and it can match the actual conditions of wastewater and reduce the requirement for carbon sources [6,11,20]. Furthermore, the A2/O process has temporal changes in redox conditions that create an environment favorable for sustaining sulfide for extended time periods, and previous researchers have reported sulfide-related corrosion in the A2/O process [14]. Accordingly, we hypothesized that the N removal under a low COD/TN ratio in the YXM WWTP was due to mixotrophic denitrification facilitated by the sulfur cycle.

In this study, we sought to reveal i) whether mixotrophic denitrification facilitated by sulfur-based autotrophic denitrification could be developed in full-scale WWTPs using A2/O through acclimation to low COD/TN and high sulfate concentrations and ii) the underlying microbial and genetic mechanisms of mixotrophic denitrification in full-scale A2/O WWTPs. Until now, A2/O process, i.e., anoxic denitrification followed by aerobic oxidation of organic and N, and then recycling of nitrate back to the anoxic reactor for denitrification to N_2_ is still the most common mainstream biological N and phosphorus removal process used in full-scale WWTPs [2]. Despite innovations leading to energy-efficient N management, the improvement of existing WWTPs to fulfil stringent N regulation will be increasingly valuable [14]. Understanding the underlying mechanisms of mixotrophic denitrification in full-scale A2/O WWTPs will help to design and reform existing full-scale A2/O WWTPs to improve the N removal efficiency, cost savings and sludge minimization.

## Materials and methods

### Description of the YXM WWTP

The WWTP built in Yixing, Jiangsu, China (YXM WWTP), was designed to receive 60% local urban sewage and 40% industrial wastewater. The process flow of the YXM WWTP, consisting of pre-anoxic (PRAN), anaerobic (ANA), anoxic (AN), and aerobic (AE) units, followed by post-anoxic (POAN) and post-aerobic (POA) units (S1 Fig and S1 Table), was described in Chen et al. [19]. During the operation, the external cycling liquid was returned to PRAN, and the internal cycling liquid was returned to the AN unit [19]. The influent was distributed to PRAN and ANA at a ratio of 3:7. Stable operation of the YXM WWTP began after nearly one year of operation [19]. After one year of stable operation, mixed liquid samples were collected from PRAN, ANA, AN, and POAN units.

### Analytical methods

The mixed liquid was sampled from the PRAN, ANA, AN, and POAN units and filtered through a millipore filter (0.45 μm), and the filtrates were analyzed for TN, NH_4_^+^-N, NO_2_^-^-N, NO_3_^-^-N, COD and TP using standard methods of the National Environmental Bureau [21]. Sulfate (SO_4_^2-^-S) was analyzed using an ion chromatography method with a conductivity detector (DIONEX-100, IonPac AS9-HC analytical column). The TDS (total dissolved sulfide, including H_2_S, HS^-^, and S^2-^) was measured using an iodometric method [22]. The subsamples used for TDS analysis were supplemented with 0.1% v/v of 10 mol/L NaOH to prevent H_2_S from escaping. All samples were collected and run in triplicate.

The TN removal efficiency, ΔCOD and ΔTN were calculated as follows:

$$TN\;removal\;efficiency=\left(TN_{inlet}‐TN_{outlet}\right)/TN_{inlet}$$ (1)

$$\Delta COD=COD_{inlet}‐COD_{outlet}$$ (2)

$$\Delta TN=TN_{inlet}‐TN_{outlet}$$ (3)

The relationships of COD/TN and ΔCOD/ΔTN with TN removal efficiency were analyzed with linear regression in GraphPad Prism 7.0.

### Nucleic acid extraction and cDNA synthesis

Mixed active sludge (AS) liquid (100 mL) was collected from the PRAN, ANA, AN, and POAN units and then centrifuged at 10,000 g for 10 min to collect the AS pellet. Total DNA was extracted from 100 mg of the AS pellet using the FastDNA spin kit for soil and the FastPrep instrument (both from MP Biomedicals, Santa Ana, CA) following the manufacturer’s instructions. The concentration and purity of the extracted DNA were quantified micro-spectrophotometrically (NanoDrop® ND-1000, NanoDrop Technologies, Wilmington, DE, USA).

Total RNA was extracted from 100 mg of the AS pellet using the FastRNA Pro Soil-Direct kit (MP Biomedicals) according to the manufacturer’s instructions. The extracted RNA was immediately reverse transcribed into cDNA using the PrimeScript^TM^ RT reagent kit (Takara, Dalian, China) following the manufacturer’s protocol. DNA and cDNA were stored at -80 ^o^C before further analysis.

### DNA library construction and metagenomic sequencing

The DNA library was constructed following the manufacturer’s instructions (Illumina). Briefly, the DNA was sheared using an M220 Focused-ultrasonicator^TM^ (Covaris Inc., Woburn, MA, USA), and fragments of ~300 bp were extracted for paired-end library construction. The DNA fragments were processed by end repair, A-tailing, adapter ligation, DNA size selection, PCR, and PCR-product purification according to the Illumina TruSeq DNA sample prep v2 guide. The average insert size of the library was 350 bp. Paired-end sequencing (2 × 100 bp) was performed on an Illumina genome analyzer (HiSeq2000, Illumina) at Majorbio Bio-Pharm Technology Co., Ltd. (Shanghai, China) using the TruSeq PE cluster kit v3-cBot-HS and the TruSeq SBS kit v3-HS according to the manufacturer’s instructions (Illumina). All original metagenomic sequences were archived at the NCBI Sequence Read Archive (SRA) under accession SRP140747.

### Taxonomic and functional annotation analyses

Merged long-read sequence sets were blasted for sequence matching using the NR (nonredundant) database. The BLASTX alignments were further processed using MEGAN5 [23] to statistically analyze the abundance of each taxon. MEGAN (MEta Genome ANalyzer) software uses a homology-matching algorithm to generate a phylogenetic tree based on the GenBank taxonomic database.

The protein-coding reads were annotated in two steps: 1) the predicted open reading frame (ORF) was subjected to Rap-search (version 2.0) [24] against the entries in the NCBI NR database, with an E value cutoff of 1e-5; 2) the reads were mapped to the ORFs using the bow-tie program [25], allowing two mismatches. The number of reads mapped to each ORF was counted using the SAM tools package [26] and used to quantify ORF abundance within the metagenome. MEGAN5 [23] was used to parse the tabular outputs of BLASTN and Rap-search into various taxonomic and functional (SEED) levels. For comparison purposes, all distributions were normalized as a function of the number of annotated sequences/genes.

KEGG (Kyoto Encyclopedia of Genes and Genomes) Orthology (KO) annotation was conducted in a BLAST search (v2.2.25) against the KEGG database (http://www.genome.jp/keeg/). Hierarchical clustering analysis of the KO annotation results obtained for the different samples were carried out using Hierarchical Clustering Explorer.

### Taxonomic analysis of the denitrification gene sequences

MG-RAST was used to assign the *narG*, *napA*, *nirS*, *nirK*, *norB*, and *nosZ* genes to specific bacterial genera by BLASTX against the entries in the NCBI-NR database, with an E-value cut off of 1e-5. The BLASTX results were visualized using MEGAN (http://ab.inf.uni-tuebingen.de/software/megan/) at a threshold of a bit score > 50 [27].

### Gene expression activity analysis using quantitative PCR (qPCR) and reverse transcription qPCR (RT-qPCR)

SYBR Green I qPCR was performed to estimate the abundance and expression of the N-cycling functional genes (*amoA*, *narG*, *napA*, *nirS*, *nirK*, *norB*, and *nosZ*) by using DNA and cDNA as templates. The primer pairs and programs used in the qPCR are listed in S2 Table. Standard plasmids of the genes of interest were prepared as described in a previous study [28]. Each reaction was performed in triplicate.

### Statistical analysis

SPSS 19.0 was used to calculate the Spearman index and to perform the correlation analysis. A *p*-value < 0.05 was considered to indicate statistical significance. Data were expressed as the mean ± SD.

## Results and discussion

### Sulfur-cycling facilitated nitrogen removal

**Fig 1:**
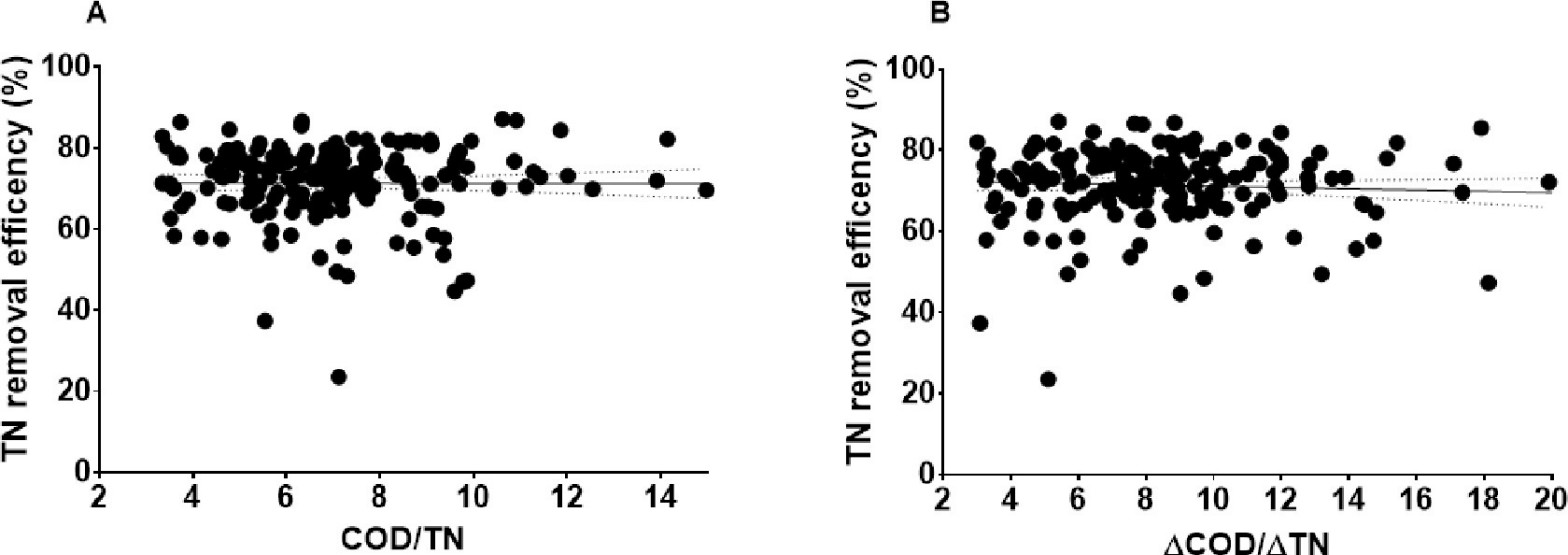
The relationship of COD/TN (A) and ΔCOD/ΔTN (B) with TN removal efficiency.

The influent of the YXM WWTP was characterized by a low ratio of COD/TN (< 6) [19] and a high sulfate concentration (> 100 mg SO_4_^2-^-S L^-1^). Additionally, the ratio of BOD/COD in the YXM WWTP was only approximately 0.35. However, the effluent quality could meet the first class A criteria of effluent discharge, and the average removal efficiency of COD, TN and NH_4_^+^-N could reach 83%, 72.4%, and 98.6%, respectively [19]. Both COD/TN and ΔCOD/ΔTN showed no significant relationship with TN removal efficiency (*p* > 0.05, Fig 1), which indicated that the ratio of COD/TN did not significantly influence the N removal processes in the YXM WWTP.

In the PRAN unit, NO_3_^-^-N decreased from 7.41 ± 0.44 mg L^-1^ in the influent to 1.03 ± 0.25 mg L^-1^ in the effluent, and the concentration of NO_3_^-^-N continued to decrease to 0.41 ± 0.08 mg L^-1^ in the ANA unit effluent (Table 1). Meanwhile, approximately 12 mg L^-1^ SO_4_^2-^-S was reduced, and 10 mg L^-1^ TDS was produced in the ANA unit. In AN, the main denitrification unit in the A2/O process received 400% nitrate-rich internal influx from the AO unit (S1 Fig), and approximately 17 mg L^-1^ COD was removed, which at most corresponded to approximately 7 mg L^-1^ TN removal. Previous studies reported that 2.86 mg L^-1^ BOD was needed for complete denitrification of 1 mg L^-1^ nitrate [29]. However, approximately 8 mg L^-1^ TN was removed in AN, which indicated that heterotrophic denitrification alone could not meet the need for N removal even though all of the consumed COD was used for denitrification. In addition to COD reduction, we also found that approximately 9 mg L^-1^ TDS was oxidized to SO_4_^2-^-S in AN, which stoichiometrically corresponded to approximately 6 mg L^-1^ TN removal following the equation of 5HS^-^ + 8NO_3_^-^ + 3H^+^ → 5SO_4_^2-^ + 4N_2_ + 4H_2_O [30]. Thus, the combined data showed that the internal sulfur cycle of sulfate reduction and re-oxidation did occur in the A2/O system, and sulfur-based autotrophic denitrification could be a supplement to the integrated denitrification capacity.

**Table 1:** Performance of the PRAN, ANA, AN and POAN chambers.

Parameter	PRAN influent	PRAN effluent	ANA effluent	AN effluent	POAN effluent
COD (mg L^-1^)	74.23 ± 5.01	65.27 ± 3.02	77.06 ± 2.34	60.63 ± 3.03	38.65 ± 3.56
TN (mg L^-1^)	21.2 ± 1.55	18.85 ± 0.98	18.2 ± 0.39	10.38 ± 1.61	9.76 ± 0.24
NH_4_^+^-N (mg L^-1^)	9.38 ± 0.37	13.22 ± 0.56	15.09 ± 0.48	7.95 ± 0.21	0.67 ± 0.08
NO_3_^-^-N (mg L^-1^)	7.41 ± 0.44	1.03 ± 0.25	0.41 ± 0.08	1.48 ± 0.15	8.56 ± 0.31
SO_4_^2-^-S (mg L^-1^)	110.02 ± 10.08	109.35 ± 11.13	98.65 ± 10.31	107.22 ± 15.34	109.26 ± 13.06
TDS (mg L^-1^)			11.13 ± 1.51	2.33 ± 0.32	< 0.1
DO		0.3–0.5	0.01–0.1	0.1–0.5	-0.4–0.7

PRAN: Pre-anoxic unit, ANA: Anaerobic unit, AN: Anoxic unit, POAN: Post-anoxic unit.

Simultaneous sulfur-based autotrophic and heterotrophic denitrification has been reported to be achieved in pilot and full-scale bioreactors [6,31,32]. Wu et al. [16] reported that a high sulfate-to-COD ratio (> 1.25 mg SO_4_^2-^/mg COD) facilitated the development of sulfur-based autotrophic denitrification. Accordingly, the average concentration of SO_4_^2-^-S was over 100 mg L^-1^ in the inlet of the YXM WWTP, which was high enough to provide sufficient sulfur through sulfate reduction. In addition, the A2/O process has varying redox environments, including anaerobic and anoxic zones, which are favorable for sulfate reduction and sulfur oxidation. Normally, the redox potential needed for sulfate reduction is lower than that needed for denitrification, and NO_3_^-^-N can interfere with sulfate reduction. In the YXM WWTP, NO_3_^-^-N and DO were consumed in PRAN before entering the ANA unit (Table 1), which facilitated the establishment of an anaerobic environment suitable for sulfate reduction in ANA [33]. Furthermore, the floc micro-environment might also play an important role in sulfur-based denitrification in the A2/O process. AS flocs are partially penetrated by oxygen, and the outer portion of the flocs is aerobic, while the inner portion of the flocs will be anoxic and/or anaerobic [34], which could provide a critical micro-environment for the harmonious coexistence of SRB, heterotrophic denitrifiers and sulfur-based autotrophic denitrifiers.

### Overview of the microbial community and key bacteria involved in sulfur cycling facilitated nitrogen removal

**Fig 2:**
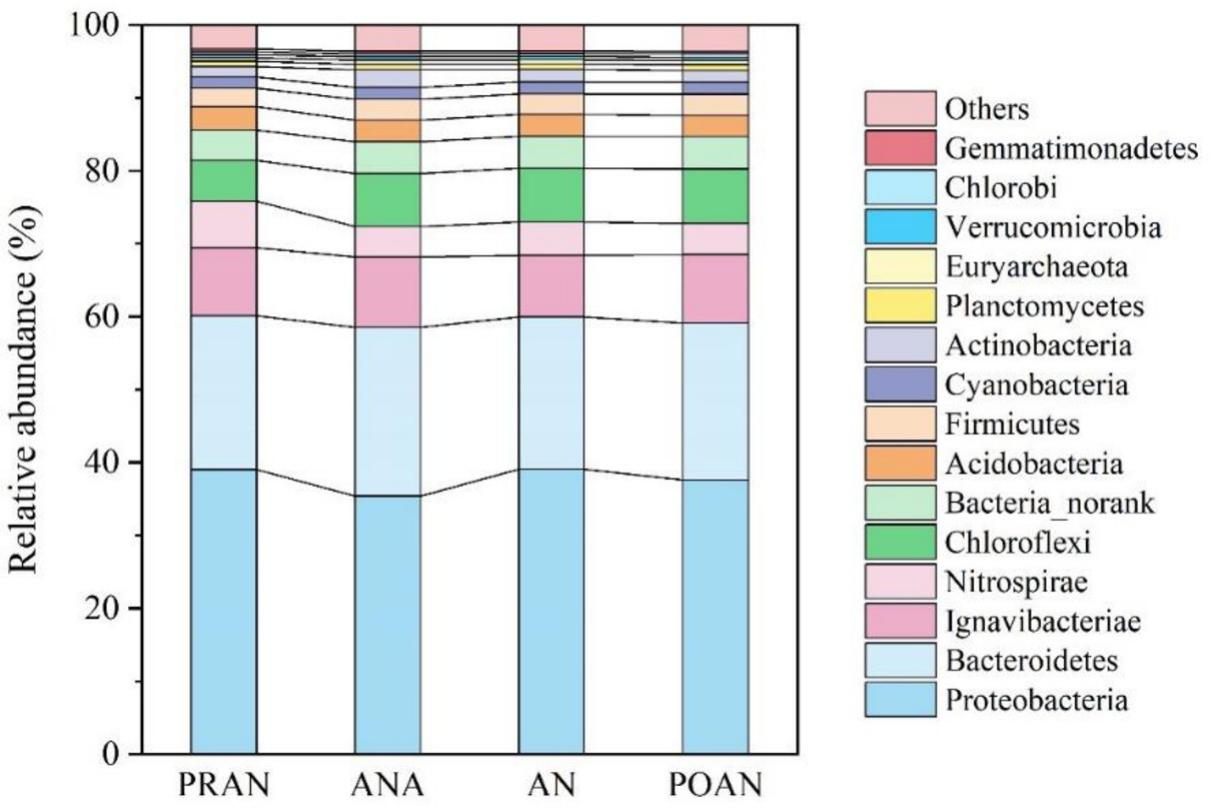
Bacterial community composition at the phylum level, determined using metagenomic sequencing, in the four denitrification units.

To reveal the underlying microbial and genomic mechanisms of the observed denitrification under low COD/TN, the metagenome of the AS in the YXM WWTP was analyzed. The AS microbial communities were composed of 86 phyla and over 1,700 genera. Bacteria (5,612,582–6,168,910 reads, 98.22%) dominated the AS microbial communities, followed by Archaea (36,272–45,988 reads, 0.72%), Eukarya (13,566–17,280 reads, 0.27%), and viruses (25,632–42,440 reads, 0.80%). For bacteria dominant in AS microbial communities, we focused our analysis on the domain of bacteria. Similar to other full-scale N removal WWTPs [19,35], Proteobacteria (35.2%-38.8%), Bacteroidetes (20.8%-23.0%), Ignavibacteriae (8.4%-9.6%), Nitrospirae (4.1%-6.3%), Chloroflexi (5.6%-7.4%), Acidobacteria (2.9%-3.2%), and Firmicutes (2.5%-2.9%) were the dominant bacterial phyla (Fig 2). At the class level, β-proteobacteria was the most dominant class, accounting for approximately 20.64%-23.25% of the total bacterial reads, much more than other Proteobacteria. The dominance of β-proteobacteria in sulfur-based N removal AS bacterial communities was also reported in other metagenomics studies [15,35].

**Fig 3:**
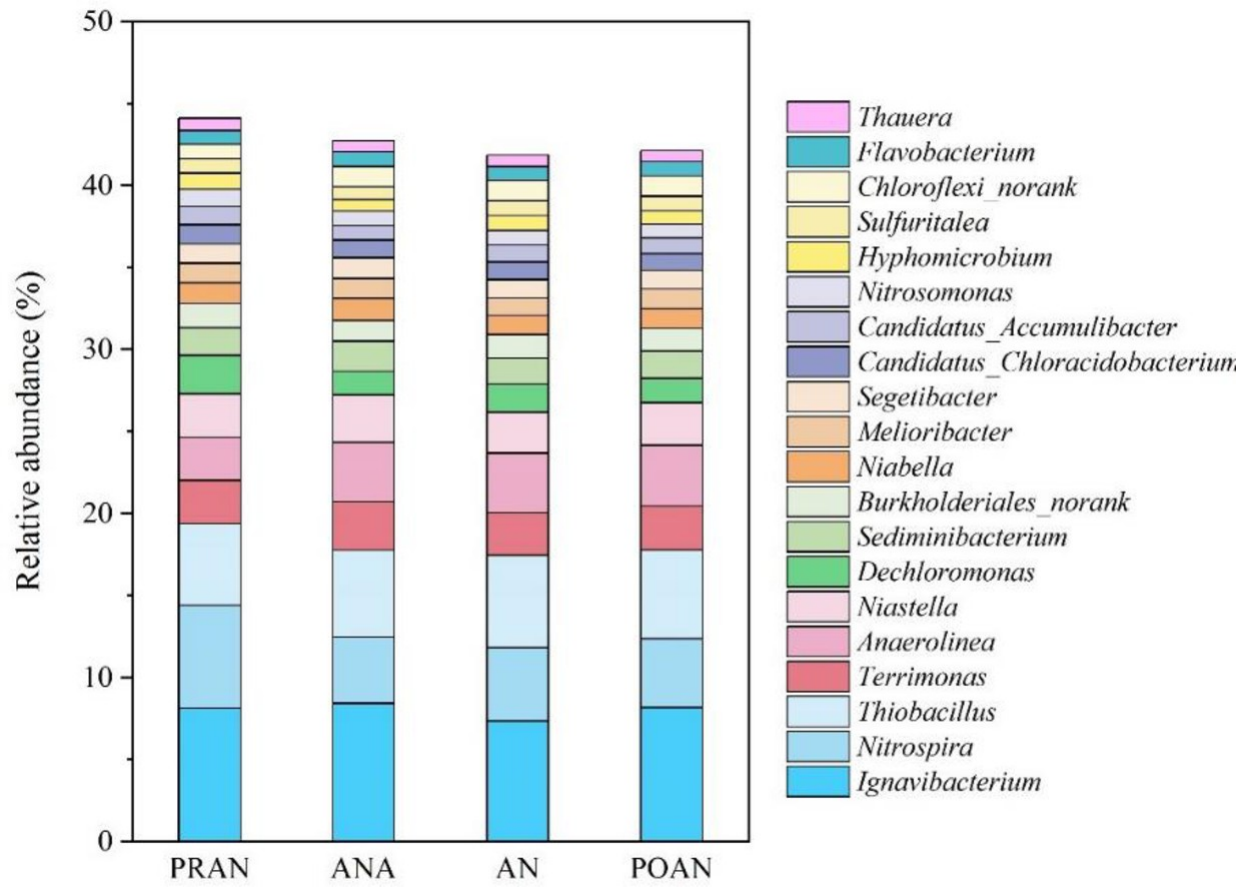
Bacterial community composition at the genus level in the four denitrification units.

At the genus level, the top 20 abundant genera collectively accounted for 42.1–44.1% of the total bacterial reads (Fig 3). Many members among the top 20 genera were associated with nitrification and sulfur-based denitrification. *Nitrospira* (4.0%-6.3%) and *Nitrosomonas* (0.8%-1.1%), typical nitrifiers, also dominated in other AS communities [19,35]. *Ignavibacterium* (7.3%-8.4%) was the most dominant genus in the AS community, which was associated with sulfur-based autotrophic denitrifying processes, converting NO_2_^-^ and NO to N_2_ [36,37]. *Thiobacillus* (4.9%-5.6%) was the third most abundant taxon, which was previously reported to be the dominant taxon in sulfur-based autotrophic denitrification bioreactors and could also cooperate with heterotrophic denitrifiers in heterotrophic environments [8,11,15,32]. *Sulfuritalea* (0.80%-0.91%) and *Thauera* (0.65%-0.76%) were also found to act as sulfur-driven autotrophic denitrifiers [13]. *Dechloromonas* (1.40%-2.30%), a heterotrophic denitrifier, was also found to contain sulfur oxidation genes [38].

Sulfate reduction by SRB provides the reduced S species for sulfur-assisted denitrification. The relative abundance of SRB (e.g., *Geobacter*, *Desulfobulbaceae*, *Pelobacteraceae*, and *Desulfobacteraceae*) was 1.74%-1.88% of the total bacterial reads. Unlike the mainstream sulfur-driven denitrification process [3,8,15], SRB were not the dominant taxa in the A2/O process. However, the guild of SRB showed high diversity in our study (40 genera) (S3 Table). The high diversity of SRB could facilitate the adaption and activity of SRB guilds when they went through units with different environments in A2/O facilities. In addition, *Ignavibacterium* might also act as the potential SRB because it was reported to have polysulfide, thiosulfate reductases, and tetrathionate reductases [36]. Meanwhile, *Ignavibacterium*, together with other fermenters, could provide organic acids (butyrate, lactate, fumarate) or alcohols (ethanol), which could be used by SRBs and heterotrophic denitrifiers [33,36,39]. Generally, the metagenomics data showed the co-existence of nitrifiers, anaerobic SRB, autotrophic SOB denitrifiers and heterotrophic denitrifiers in AS, which could provide a substantial microbial basis for sulfur-based mixotrophic N removal.

### Functional genes related to sulfur reduction and oxidation

**Fig 4:**
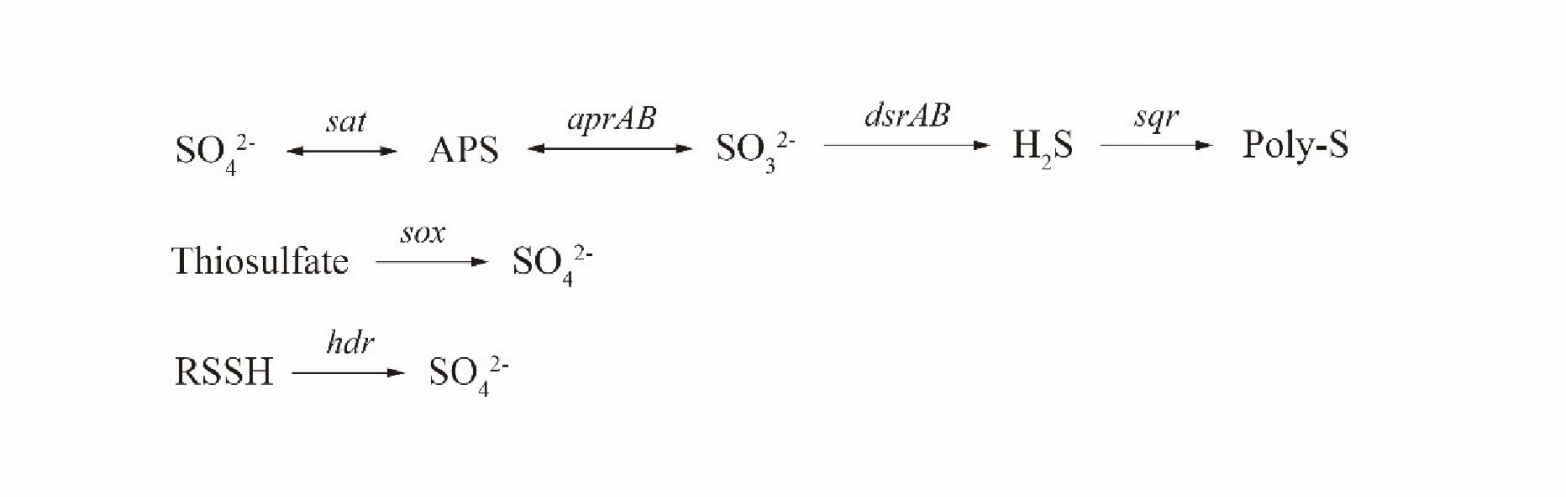
Internal cycling of sulfur through the cooperation of enzymes encoded by sulfate reduction and sulfur oxidation genes.

Sulfate reduction mediated by SRB was anticipated to provide reduced S species for electron donors in sulfur-based autotrophic denitrification, and the genes involved in dissimilatory sulfate reduction, including sulfate adenylyltransferase (*sat*), adenylylsulfate reductase (*aprAB*) and dissimilatory sulfite reductase (*dsrAB*), were found in high abundance in the AS communities of the YXM WWTP (Fig 4 and Table 2). In addition, a high abundance of the poly-S formation gene coding sulfide quinone oxidoreductase (*sqr*) was found in the AS community (Fig 4 and Table 2). Poly-S could also serve as electron and energy storage material for denitrification [8,16,40].

**Table 2:** Sulfur oxidation and sulfate reduction genes in the sludge samples.

	Gene(s)	PRAN	ANA	AN	POAN
Sulfur oxidation	*sqr*	2188	2378	2286	2474
	*soxZ*	330	320	336	348
	*soxA*	700	704	682	676
	*soxY*	702	586	676	718
	*soxB*	1022	1030	1024	1130
	*soxX*	428	426	424	452
	*hdrC*	284	316	360	358
	*hdrB*	760	822	852	800
	*hdrA*	750	838	956	912
Sulfate reduction	*dsrB*	432	502	462	504
	*dsrA*	588	626	646	694
	*sat*	4044	4560	4746	4908
	*aprB*	262	268	254	264
	*aprA*	864	802	948	944

PRAN: Pre-anoxic, ANA: Anaerobic, AN: Anoxic, POAN: Post-anoxic.

Sulfur oxidation genes, including those for sulfur oxidation multienzyme complex (*sox*) and heterodisulfide reductase (*hdr*), were detected in the AS communities (Fig 4 and Table 2). The *Sox* enzyme system oxidizes thiosulfate, which is produced by SRB or chemical oxidation of H_2_S [33,41,42]. A previous study showed that the *sox* and *hdr* genes were simultaneously expressed with denitrification genes in *Thiobacillus* and *Thauera* [13], indicating the important role of *sox* and *hdr* in sulfur-based denitrification. Therefore, the metagenomic analysis of internal sulfur cycling-related genes showed that the AS of the YXM WWTP could proceed with the internal cycling of sulfur through the cooperation of enzymes encoded by sulfate reduction and sulfur oxidation genes, which consolidated the possibility of sulfur-based autotrophic denitrification without additional TDS supplementation (Fig 4).

### Functional genes related to nitrogen transformation and taxonomic classification of denitrification genes

Of the detected functional genes related to N cycling, those related to denitrification had the highest number of hits, followed by ammonification and nitrification genes. The functional gene related to the anammox process (*hzo*, encoding hydrazine oxidoreductase) was not detected in this study, which suggested that denitrification was the main pathway of N removal in the YXM WWTP. Among the denitrification genes, the abundance of *narG* was the highest (4,320–4,660 reads), followed by *napA* (2,366–2,618 reads), *nosZ* (2,122–2,296 reads), and *norB* (1,130–1,330 reads), whereas the abundances of *nirK* (748–798 reads) and *nirS* (1,044–1,120 reads) were much lower (Table 3).

**Table 3:** The abundances of genes related to nitrogen removal.

	Gene(s)	PRAN	ANA	AN	POAN
Ammonification	*gdhA*	984	1016	1004	1110
	*ureE*	84	68	60	64
	*ureD*, *ureH*	6	12	16	8
	*ureC*	236	238	210	196
Nitrification	*pmoA-amoA*	88	106	86	74
	*pmoB-amoB*	108	98	72	134
	*hao*	536	602	550	636
Denitrification	*narG*	4320	4660	4440	4562
	*napA*	2366	2578	2486	2618
	*nosZ*	2122	2296	2138	2146
	*norB*	1130	1214	1192	1330
	*nirS*	1044	1082	1062	1120
	*nirK*	778	748	772	798
	*norC*	340	416	324	342
Dissimilatory nitrate reduction	*nirB*	1012	1094	1074	1024
	*nrfA*	606	818	718	810
Assimilatory nitrate reduction	*narB*	10	30	22	24
	*nasA*	612	624	604	628
	*nirA*	238	170	158	162

PRAN: Pre-anoxic, ANA: Anaerobic, AN: Anoxic, POAN: Post-anoxic.

**Fig 5:**
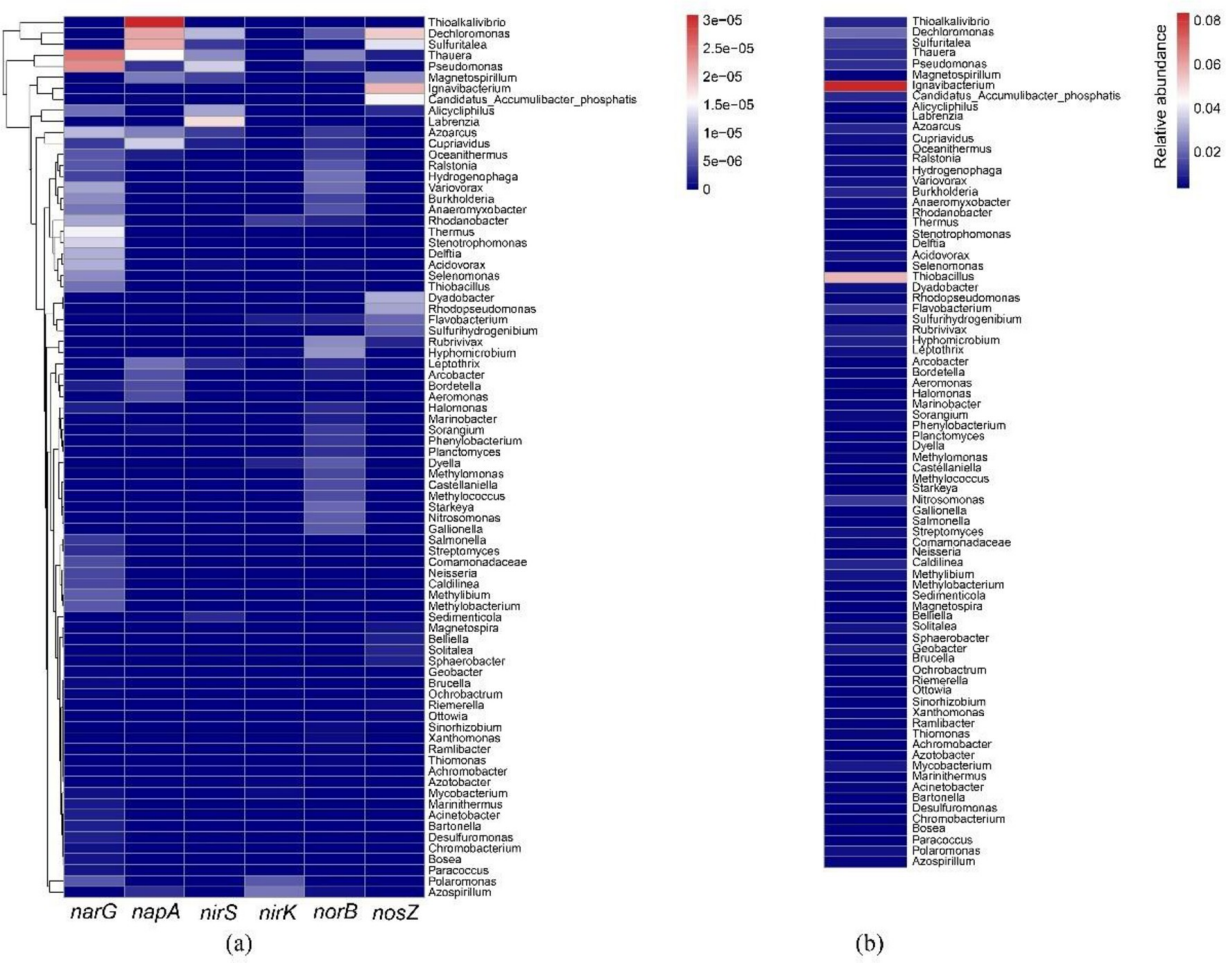
Taxonomic distribution of denitrification genes (a) and relative abundance of taxa (b).

The taxonomic origins of the denitrification genes were also analyzed. The results showed that denitrification genes were unevenly distributed in eighty genera, whereas most of them were affiliated with β-proteobacteria (Fig 5), which was consistent with the dominance of β-proteobacteria in the AS communities. The *narG* gene was mostly associated with *Thauera*, *Pseudomonas*, *Thermus*, *Azoarcus*, *Stenotrophomonas*, *Acidovorax*, *Rhodanobacter*, and *Thiobacillus*. The *napA* gene was mainly associated with *Thauera*, *Thioalkalivibrio*, *Sulfuritalea*, *Dechloromonas*, *Cupriavidus*, and *Leptothrix* (Fig 5). Among the genes required for the transformation of NO_2_^-^-N to NO, *nirS* was mainly associated with *Labrenzia*, *Pseudomonas*, *Alicycliphilus*, *Thauera*, *Sulfuritalea*, and *Dechloromonas*, while *nirK* was mainly affiliated with *Azospirillum*, *Rhodanobacter*, and *Polaromonas*. The *norB* gene was mostly associated with *Thauera*, *Rubrivivax*, *Hyphomicrobium*, *Hydrogenophaga*, and *Variovorax*. In our metagenomics data, 23 genera were found to harbor *nosZ*, predominately *Dechloromonas*, *Sulfuritalea*, and *Candidatus Accumulibacter phosphatis*. The diversity of denitrifying gene associated taxa showed that every step of denitrification was metabolized by a combination of gene products that originated from different bacterial species. Complete denitrification is achieved through the combined activity of taxonomically diverse co-occurring bacteria performing successive metabolic steps. The diverse denitrifiers catalyze N transformation at very different rates, however, the diverse denitrification consortia were suitable to adapt to varying environments in different units in the A2/O process.

The genes encoding enzymes initiating nitrate reduction, *narG* and *napA*, were mainly associated with autotrophic *Thiobacillus*, *Thioalkalivibrio*, and *Sulfuritalea* (Fig 5). Consistent with the dominance of *Thiobacillus* in the microbial communities, the results showed the important roles of autotrophic sulfur-based denitrifiers at the functional gene level. *Thauera* was associated with a high relative abundance of *the narG*, *napA*, *nirS*, *norB*, and *nosZ* genes (Fig 5). Both *Thauera* and *Thiobacillus* can use reduced sulfur as the electron donor and contribute to sulfur-driven autotrophic denitrification [12,13]. The analysis provided insight into microbial and functional gene foundations for the occurrence of sulfur-based mixotrophic denitrification in the A2/O process.

It must be noted that most of the denitrifier taxa and high abundance of the denitrification genes *narG*, *napA*, *nirS*, *norB*, and *nosZ* were associated with heterotrophic denitrifiers, such as *Dechloromonas*, *Pseudomonas*, and *Rhodanobacter*. *Dechloromonas*, which carries the *napA*, *nirS*, *norB*, and *nosZ* genes, accounted for a relatively high percentage of the denitrifiers (Fig 5). *Dechloromonas*, which was found to act as a heterotrophic sulfur oxidizing denitrifier [43], might also contain sulfur oxidation genes [38]. The results were not surprising because heterotrophic denitrification was still the main N removal pathway in the A2/O process. Notwithstanding, the coexistence of autotrophic and heterotrophic denitrifiers provided evidence that denitrification in the YXM WWTP could be a mixotrophic process.

Mixotrophic denitrification involves the use of fewer carbon sources and less sulfate production than heterotrophic or autotrophic denitrification and thus allows for a relatively good rate of nitrate removal over a long period of time while achieving efficient COD and N removal [44]. Notably, the mixotrophic denitrification process could achieve high efficiency of NH_4_^+^-N removal, as indicated in our study and others [43]. We should keep in mind that electron donors significantly impact the microbial community structure and composition [32]. Our previous study also showed that the addition of external carbon source could improve the efficiency of TN removal, however, the external carbon source elevated the proportion of heterotrophic denitrifiers in the AS communities and incurred feedback for more carbon sources [45]. Additional exploration of the communities in sulfur-based mixotrophic denitrification, with clearer variations in performance parameters, as well as the interactions between “*Thiobacillus*” and heterotrophic denitrifiers in A2/O, will facilitate the development of low-cost and more flexible denitrification processes.

### Activities of nitrification and denitrification genes

Although SONB can tolerate a certain concentration of sulfide, a high concentration of sulfide can inhibit their denitrification activity [12]. A previous study also showed that the activities of nitrification were inhibited by sulfide because ammonia oxidizers were sensitive to sulfide [14]. To evaluate the influence of TDS on mixotrophic denitrification in the YXM WWTP, the expression activities of functional genes related to N removal were analyzed using RT-qPCR.

**Fig 6:**
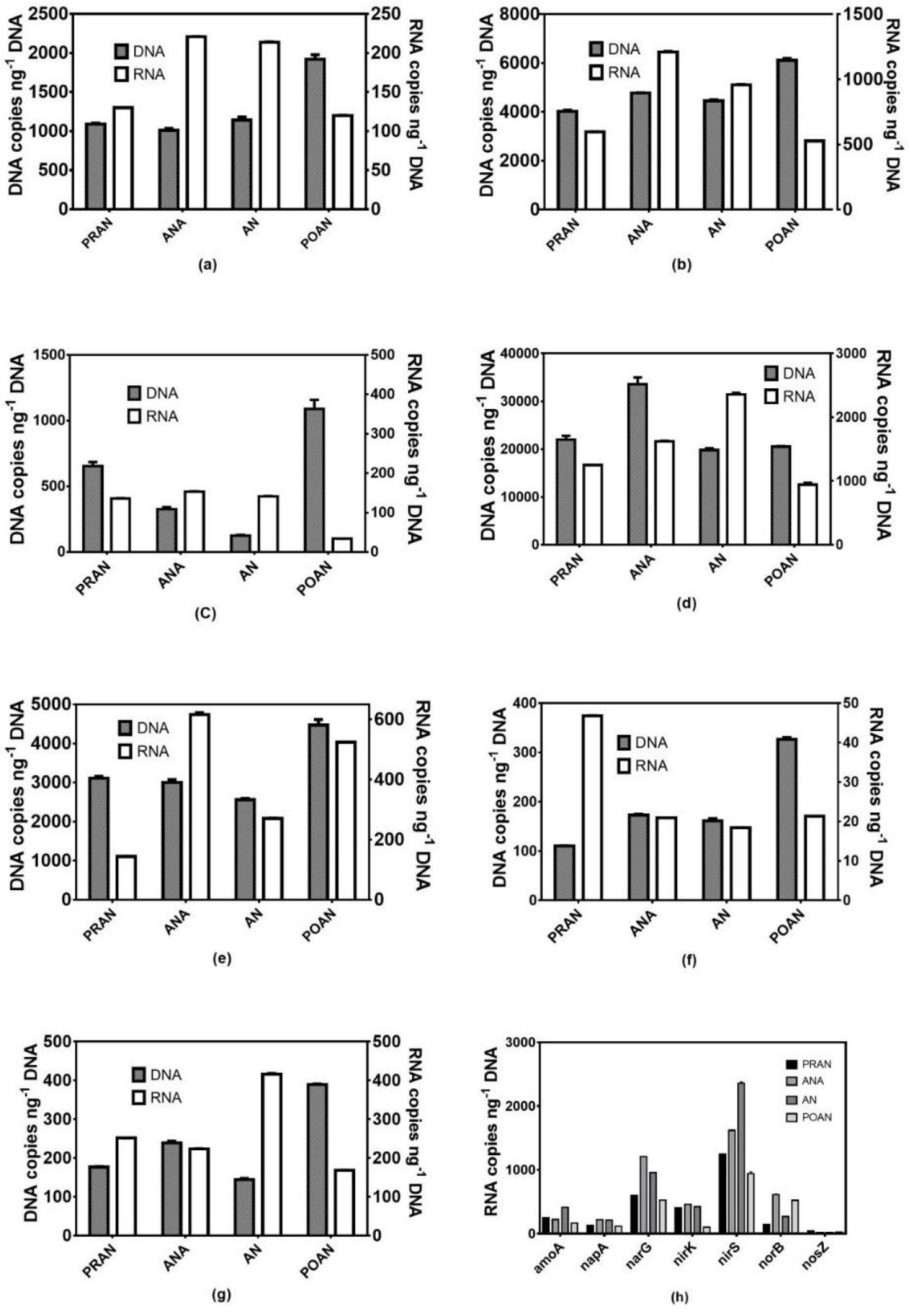
Quantitative PCR analysis of the activity of nitrifying and denitrifying bacteria based on the abundance (DNA) and expression (RNA) of nitrogen-associated genes in the four denitrification units, a: *napA*; b: *narG*; c: *nirK*; d: *nirS*; e: *norB*; f: *nosZ*; g: *amoA*; h: the expression levels of nitrogen-related genes in the four denitrification units.

In our study, a high *amoA* mRNA/DNA ratio (S4 Table) was found in all four units (from 0.43 in POAN to 2.88 in AN), and the expression of *amoA* had no significant relationship with the content of reduced sulfur, which indicated that *amoA* expression was not inhibited by sulfide, as reported in a previous study [14]. The high-level expression of *amoA* ensured a high rate of NH_4_^+^-N transformation and subsequent denitrification in the A2/O process despite the low abundance of nitrifiers (Fig 6) [46].

In agreement with the metagenomics data, the abundance and expression of the *narG* gene were higher than those of *napA* (Fig 6). Moreover, the expression of *narG* and *napA* in ANA and AN was significantly higher than that in PRAN and POAN. The high expression of *narG* confirmed the important role of *Thiobacillus* and *Thauera* in denitrification, as *Thiobacillus* and *Thauera* were the main taxa carrying *narG* (Fig 5). We also found a higher mRNA/DNA ratio of *narG* and *napA* in the ANA chamber than in other units (S4 Table), indicating that the anaerobic environment was favorable to the expression of *narG and napA*, while *narG* and *napA* were not sensitive to TDS (Fig 6). However, the low content of nitrate and short hydraulic retention time (HRT) implied no significant denitrification in the ANA chamber.

The abundance of *nirS* was higher than that of *nirK*, and the mRNA abundance of *nirS* was 3.1- to 93.3-fold higher than that of *nirK*, indicating that *nirS* was a key player in NO_2_^-^-N reduction (Fig 6). Although among the denitrification genes, the abundance of *norB* was relatively high, its expression was low, as evidenced by its mRNA/DNA ratio, which only varied from 0.05 to 0.21. The *nosZ* gene is often used as a marker of complete denitrification [47]. The activity of *nosZ* is critical for the elimination of N_2_O in the denitrification process. Some recent studies have shown that sulfur-based autotrophic denitrification could promote the production and accumulation of N_2_O as a significant intermediate product [48]. In this study, the abundances of the *nosZ* gene and mRNA were the lowest among the denitrification genes (Fig 6). Therefore, the bioaugmentation of *nosZ*-type denitrifiers, including *Thauera* and *Candidatus Accumulibacter phosphatis*, might facilitate the enhancement of the TN removal performance of WWTPs.

The correlation of the expression of *amoA* and denitrification genes and the content of TDS was analyzed, and no significant relationship was found between them (*p* > 0.05). Our results suggested that reduced sulfur did not inhibit or stimulate the potential of N removal in the AS of the YXM WWTP. The expression pattern of functional genes might be attributed to mixotrophic denitrification using both reduced sulfur and organic compounds as electron donors, which compensate for the influence of high reduced sulfur levels on functional gene activity. In the cooperation of SRB with SONB and other heterotrophic bacteria, metabolic diversity helps to relieve the inhibition of sulfide. As discussed above, HS^-^ produced by SRB might be transformed to poly-S and thiosulfide, and these reduced S species could also be oxidized by *Thiobacillus* in denitrification.

## Conclusion

Mixotrophic denitrification was reported in the A2/O process with low COD/TN and high sulfate in the influent. Metagenomic analyses revealed the mixotrophic denitrification potential based on sulfur reduction and oxidation cycles at the level of microbial communities and functional genes. High abundances of sulfur oxidation, sulfate reduction and denitrification genes were found in our study. *Thiobacillus* dominated the microbial communities, and the *narG* gene was mainly harbored by *Thiobacillus* and *Thauera*, however, denitrification genes were enriched in heterotrophic denitrifiers with high abundance. Both autotrophic and heterotrophic denitrifiers contributed to nitrogen removal. Reduced sulfur showed no inhibition of the expression of nitrifying and denitrifying genes. Further investigation aimed at manipulating the denitrification process will help to further identify the key autotrophic denitrification metabolism pathways involved in nitrogen removal and the important factors related to denitrification under different conditions.

## Supporting information

S1 Fig The detailed scheme figure of each reactor unit in a pilot A2/O bioreactor. (DOCX)

S1 Table Design parameters of the municipal wastewater treatment plant in Yixing, Jiangsu, China (YXM WWTP). (DOCX)

S2 Table Primers and programs of the target genes in the quantitative PCR analysis. (DOCX)

S3 Table Relative abundance of sulfate reducing bacteria guild. (DOCX)

S4 Table The mRNA/DNA ratio of nitrogen transformation genes. (DOCX)

S1 Dataset (XLSX)
