# Supplementary figures and images for: Metagenomic insights into mixotrophic denitrification facilitated nitrogen removal in a full-scale A2/O wastewater treatment plant
Source: PLoS One. 2021 Apr 15;16(4):e0250283. doi: 10.1371/journal.pone.0250283 (PMC8049308; doi:10.1371/journal.pone.0250283)

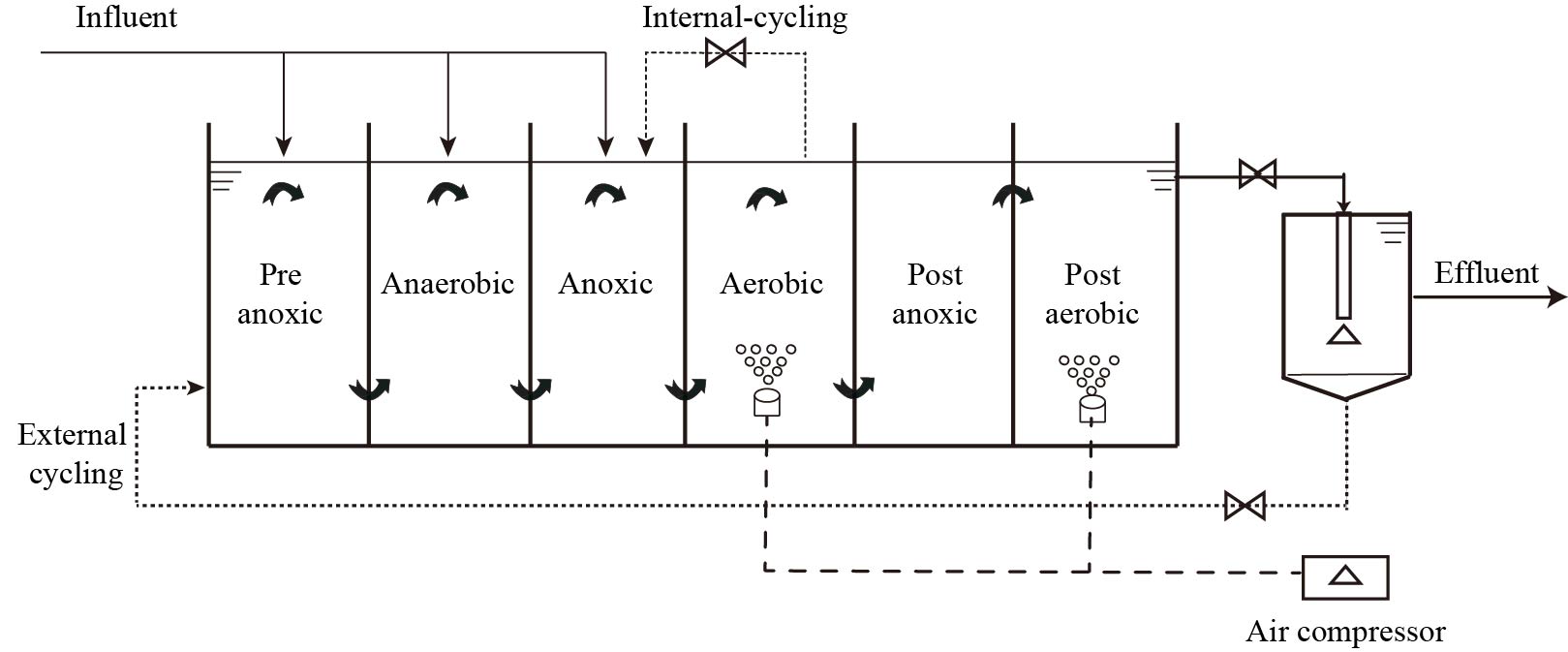


**S1 Fig. The detailed scheme figure of each reactor unit in a pilot A2/O bioreactor.**

Supplement: S1 Fig — (DOCX) [file pone.0250283.s001.docx]
