# Supplementary material for: Metagenomic insights into mixotrophic denitrification facilitated nitrogen removal in a full-scale A2/O wastewater treatment plant
Source: PLoS One. 2021 Apr 15;16(4):e0250283. doi: 10.1371/journal.pone.0250283 (PMC8049308; doi:10.1371/journal.pone.0250283)
Supplement: S1 Table — (DOCX) [file pone.0250283.s002.docx]

**Supporting Information for**

**Metagenomic insights into mixotrophic denitrification facilitated nitrogen removal in a full-scale A2/O wastewater treatment plant**

Shulei Liu, Yasong Chen, Lin Xiao*

**Affiliation**

School of the Environment, State Key Laboratory for Pollution Control and Resource Reuse (SKL-PCRR), Nanjing University, Nanjing, China

*Corresponding author: Lin Xiao

E-mail: xiaolin@nju.edu.cn

**S1 Table.** **Design parameters of the municipal wastewater treatment plant in Yixing, Jiangsu, China (YXM WWTP).**

|  | **Parameter** |
| --- | --- |
| **PRAN** | 4884 m^3^ HRT=1.02 h |
| **ANA** | 7260 m^3^ HRT=1.52 h |
| **AN** | 46042 m^3^ HRT=9.61 h |
| **AE** | 52536 m^3^ HRT=10.99 h |
| **POAN** | 8721 m^3^ HRT=1.82 h |
| **POA** | 2904 m^3^ HRT=0.61 h |
| **HRT** | 25.53 h |
| **SRT** | 22 days |
| **F/M** | 0.03 kg BOD_5_/kg MLSS·day |
| **MLSS** | 3.5 g/L |

PRAN: pre-anoxic, ANA: anaerobic, AN: anoxic, AE: aerobic, POAN: post-anoxic, POA: post-aerobic, HRT: hydraulic retention time, SRT: solids retention time, F/M: food-to-microorganisms ratio, MLSS: mixed liquor suspended solids
