# Supplementary material for: Metagenomic insights into mixotrophic denitrification facilitated nitrogen removal in a full-scale A2/O wastewater treatment plant
Source: PLoS One. 2021 Apr 15;16(4):e0250283. doi: 10.1371/journal.pone.0250283 (PMC8049308; doi:10.1371/journal.pone.0250283)
Supplement: S2 Table — (DOCX) [file pone.0250283.s003.docx]

**S2 Table. Primers and programs of the target genes in the quantitative PCR analysis.**

| Target gene | Primers | Program |
| --- | --- | --- |
| Bacterial 16S rRNA | 338F: ACTCCTACGGGAGGCAGCAG  518R: ATTACCGCGGCTGCTGG | Pre-denaturation at 95°C for 5 min, denaturation at 95°C for 15 s, annealing at 60°C for 1 min, and extension at 72°C for 1 min |
| Bacterial *amoA* | amo598f: GAATATGTTCGCCTGATTG  amo718r: CAAAGTACCACCATACGCAG | Pre-denaturation at 95°C for 5 min, denaturation at 95°C for 15 s, annealing at 56°C for 45 s, and extension at 72°C for 30 s |
| *narG* | 1960m2f: TAYGTSGGGCAGGARAAACTG  2050m2r: CGTAGAAGAAGCTGGTGCTGTT | Pre-denaturation at 95°C for 5 min, denaturation at 95°C for 15 s, annealing at 58°C for 45 s, and extension at 72°C for 30 s |
| *napA* | V17F: TGGACVATGGGYTTYAAYC  4R: ACYTCRCGHGCVGTRCCRCA | Pre-denaturation at 95°C for 5 min, denaturation at 95°C for 15 s, annealing at 56°C for 45 s, and extension at 72°C for 30 s |
| *nirK* | 583F: TCATGGTGCTGCCGCGKGACGG  909R: GAACTTGCCGGTKGCCCAGAC | Pre-denaturation at 95°C for 5 min, denaturation at 95°C for 15 s, annealing at 64°C for 40 s, and extension at 72°C for 30 s |
| *nirS* | Cd3aF: AACGYSAAGGARACSGG  R3cd: GASTTCGGRTGSGTCTTSAYGAA | Pre-denaturation at 95°C for 5 min, denaturation at 95°C for 15 s, annealing at 57°C for 30 s, and extension at 72°C for 30 s |
| *norB* | 2F: GGNCAYCARGGNTAYGA  5R: ACCCANAGRTGNACNACCCACCA | Pre-denaturation at 95°C for 5 min, denaturation at 95°C for 15 s, annealing at 54°C for 30 s, and extension at 72°C for 30 s |
| *nosZ* | 1527F: CGCTGTTCHTCGACAGYCA  1773R: ATRTCGATCARCTGBTCGTT | Pre-denaturation at 95°C for 5 min, denaturation at 95°C for 15 s, annealing at 54°C for 50 s, and extension at 72°C for 30 s |
