# Supplementary material for: Metagenomic insights into mixotrophic denitrification facilitated nitrogen removal in a full-scale A2/O wastewater treatment plant
Source: PLoS One. 2021 Apr 15;16(4):e0250283. doi: 10.1371/journal.pone.0250283 (PMC8049308; doi:10.1371/journal.pone.0250283)
Supplement: S3 Table — (DOCX) [file pone.0250283.s004.docx]

**S3 Table. Relative abundance of sulfate reducing bacteria guild.**

| Genus | Relative abundance (%) | | | |
| --- | --- | --- | --- | --- |
|  | PRAN | ANA | AN | POAN |
| *Desulfarculus* | 0.016571 | 0.017199 | 0.019664 | 0.016832 |
| *Desulfatibacillum* | 0.032088 | 0.035554 | 0.03827 | 0.035732 |
| *Desulfatirhabdium* | 0.028858 | 0.03196 | 0.029223 | 0.031214 |
| *Desulfitibacter* | 0.014148 | 0.020504 | 0.017684 | 0.017914 |
| *Desulfitobacterium* | 0.03451 | 0.038763 | 0.039943 | 0.039614 |
| *Desulfobacca* | 0.023592 | 0.022847 | 0.024853 | 0.023959 |
| *Desulfobacter* | 0.02784 | 0.032024 | 0.031237 | 0.031119 |
| *Desulfobacterium* | 0.094543 | 0.09158 | 0.096579 | 0.097969 |
| *Desulfobacula* | 0.013797 | 0.015402 | 0.017513 | 0.017182 |
| *Desulfobulbus* | 0.056558 | 0.054422 | 0.058582 | 0.055682 |
| *Desulfocapsa* | 0.015693 | 0.016493 | 0.016387 | 0.015591 |
| *Desulfococcus* | 0.090647 | 0.089976 | 0.094975 | 0.092719 |
| *Desulfocurvus* | 0.004213 | 0.004428 | 0.004438 | 0.003882 |
| *Desulfofustis* | 0.011831 | 0.011359 | 0.011949 | 0.012218 |
| *Desulfohalobium* | 0.010673 | 0.012996 | 0.0127 | 0.013077 |
| *Desulfomicrobium* | 0.019379 | 0.01736 | 0.020279 | 0.020587 |
| *Desulfomonile* | 0.059998 | 0.073097 | 0.068517 | 0.068823 |
| *Desulfonatronospira* | 0.010041 | 0.010204 | 0.011163 | 0.011836 |
| *Desulfonatronovibrio* | 0.003054 | 0.002631 | 0.003107 | 0.00245 |
| *Desulfonatronum* | 0.017483 | 0.016846 | 0.017001 | 0.01715 |
| *Desulfonauticus* | 0.00158 | 0.001925 | 0.001878 | 0.002132 |
| *Desulforegula* | 0.011761 | 0.012579 | 0.012153 | 0.011232 |
| *Desulfosarcina* | 0.018221 | 0.021018 | 0.019869 | 0.020141 |
| *Desulfospira* | 0.026436 | 0.027821 | 0.029223 | 0.028096 |
| *Desulfosporosinus* | 0.047324 | 0.056443 | 0.053325 | 0.056764 |
| *Desulfotalea* | 0.004072 | 0.005519 | 0.005257 | 0.005377 |
| *Desulfotignum* | 0.012568 | 0.015049 | 0.016489 | 0.018009 |
| *Desulfotomaculum* | 0.136426 | 0.155147 | 0.155605 | 0.160429 |
| *Desulfovermiculus* | 0.00811 | 0.007733 | 0.007306 | 0.008082 |
| *Desulfovibrio* | 0.199163 | 0.211976 | 0.222381 | 0.231671 |
| *Desulfovirgula* | 0.022504 | 0.030644 | 0.02994 | 0.030037 |
| *Desulfurella* | 0.002493 | 0.003113 | 0.002526 | 0.002864 |
| *Desulfurispirillum* | 0.010041 | 0.006739 | 0.007237 | 0.006873 |
| *Desulfurispora* | 0.006284 | 0.006065 | 0.006896 | 0.0056 |
| *Desulfurivibrio* | 0.012568 | 0.012803 | 0.012734 | 0.013523 |
| *Desulfurobacterium* | 0.012814 | 0.011809 | 0.012666 | 0.012505 |
| *Desulfurococcus* | 0.000316 | 0.000513 | 0.00058 | 0.000445 |
| *Desulfuromonas* | 0.085872 | 0.083494 | 0.083401 | 0.086292 |
| *Dethiobacter* | 0.00811 | 0.009145 | 0.009491 | 0.008846 |
| *Dethiosulfovibrio* | 0.000491 | 0.000481 | 0.000205 | 0.000286 |
| Total | 1.21267 | 1.295665 | 1.323226 | 1.334755 |
