# Supplementary material for: Metagenomic insights into mixotrophic denitrification facilitated nitrogen removal in a full-scale A2/O wastewater treatment plant
Source: PLoS One. 2021 Apr 15;16(4):e0250283. doi: 10.1371/journal.pone.0250283 (PMC8049308; doi:10.1371/journal.pone.0250283)
Supplement: S4 Table — (DOCX) [file pone.0250283.s005.docx]

**S4 Table.** **The mRNA/DNA ratio of nitrogen transformation genes.**

|  | *amoA* | *napA* | *narG* | *nirK* | *nirS* | *norB* | *nosZ* |
| --- | --- | --- | --- | --- | --- | --- | --- |
| PRAN | 1.43 | 0.12 | 0.15 | 0.62 | 0.06 | 0.05 | 0.43 |
| ANA | 0.94 | 0.22 | 0.25 | 1.41 | 0.05 | 0.21 | 0.12 |
| AN | 2.88 | 0.19 | 0.22 | 3.38 | 0.12 | 0.11 | 0.11 |
| POAN | 0.43 | 0.06 | 0.09 | 0.09 | 0.46 | 0.12 | 0.07 |
